# Indonesian parental knowledge, attitudes, and sources of information regarding pediatric space maintainers: a cross-sectional questionnaire-based study

**DOI:** 10.7717/peerj.20363

**Published:** 2026-01-05

**Authors:** Aishwarya Kumbhar, Bhagyashree Thakur, Dian Agustin Wahjuningrum, Suraj Arora, Krishna Prasad Shetty, Alexander Maniangat Luke, Mohmed Isaqali Karobari, Ajinkya M. Pawar

**Affiliations:** 1 Department of Conservative Dentistry and Endodontics, Nair Hospital Dental College, Mumbai, Maharashtra, India; 2 Department of Conservative Dentistry, Faculty of Dental Medicine, Universitas Airlangga, Surabaya, Indonesia; 3 Department of Dentistry, Division of District Early Intervention Centre, Thane Civil Hospital, Thane, Maharashtra, India; 4 Department of Restorative Dental Sciences, College of Dentistry, King Khalid University, Abha, Saudi Arabia; 5 Department of Clinical Science, College of Dentistry, Ajman University, Al-Jurf, Ajman, United Arab Emirates; 6 Centre of Medical and Bio-allied Health Science Research, Ajman University, Al-Jurf, Ajman, United Arab Emirates; 7 Department of Conservative Dentistry and Endodontics, Manipal College of Dental Science, MAHE Manipal, Karnataka, India; 8 Department of Restorative Dentistry & Endodontics, Faculty of Dentistry, University of Puthisastra, Phnom Penh, Cambodia; 9 Department of Conservative Dentistry and Endodontics, Saveetha Dental College and Hospital, Saveetha Institute of Medical and Technical Sciences, Saveetha University, Chennai, Tamil Nadu, India

**Keywords:** Space maintainers, Parental knowledge, Health knowledge

## Abstract

**Background:**

Space maintainers are an important part of pediatric dentistry to prevent malocclusion after premature loss of primary teeth. The use of space maintainers are limited in developing countries such as Indonesia. This survey study was conducted to determine Indonesian parents’ knowledge, attitudes, and informational sources related to paediatric space maintainers and factors influencing awareness and acceptance.

**Methods:**

A descriptive cross-sectional survey of 400 Indonesian parents was executed. Respondents completed a questionnaire distributed to them through clinics, schools, and publicly sponsored sites in social media. Descriptive statistics, chi-square tests, t-tests and multivariate logistic regression were conducted to study predictors of knowledge and attitudes.

**Results:**

Only 25% of parents indicated that they were aware of space maintainers. Urban parents (37.5%) were significantly more likely to be aware than rural parents (6.3%; *χ*^2^ = 48.35, *p* < 0.001). Urban location was an important predictor of the dependent variable of awareness, with urban parents being 8.92 times more likely to report awareness than rural parents (adjusted odds ratio (AOR) = 8.92, *p* < 0.001). At one point, awareness was related to higher comfort (*p* = 0.0013) and more favorable attitudes (*χ*^2^ = 10.88, *p* = 0.0043). Dentists were reported as the most common source of information about dental visits for parents living in urban settings (68.75%) while rural parents sourced information from community members and social media. Although awareness and attitude were correlated, for favorable perception about dental visits location was a more robust and independent predictor, (AOR for urban location = 8.27, *p* < 0.001).

**Conclusion:**

There is a marked gap in knowledge about space maintainers, among Indonesian parents, specifically in rural areas. Targeted educational efforts and proactive dental counselling are urgently needed to improve parental awareness and promote preventive orthodontic care.

## Introduction

The value of good oral health in children’s development and well-being cannot be overstated. It is essential to maintain the health of primary teeth to have optimal oral health. Primary teeth play a vital role in guiding occlusion, aesthetics, and the development of permanent dentition. Their premature loss often leads to malocclusion and costly orthodontic treatments (Han et al., 2025; Lemmers et al., 2025).

Space maintainers represent an important component of paediatric dentistry and represent an essential and beneficial option for preventing and intercepting orthodontic issues. Space maintainers are either fixed—band and loop, crown and loop, Nance appliance, distal shoe and transpalatal arch—or removable, and designed to maintain the mesiodistal space of the dental arch until the permanent successor tooth erupts (Metkar et al., 2025). The clinical evidence clearly shows the capacity of space maintainers to ameliorate the risk of malocclusion and decrease the need for orthodontic treatment later (Thakur et al., 2024). Despite their long history of use and efficacy, space maintainers, remain critically underutilized in many developing countries. Including Indonesia, this underutilization becomes a major challenge because of the under-enrolment of parents, lost time to access required paediatric dental treatment, and, lack of linkage between preventive orthodontics and routine paediatric treatment (Farlina & Maharani, 2018).

The situation in Indonesia is troubling, particularly considering it has over 270 million people, of which children are close to 30% (Hasan et al., 2024). In the 2018 Ministry of Health analysis, conducted by the Indonesian Ministry of Health, indicated that more than 93% of children aged 5–6 years had dental caries, and at least 80% of the children had untreated cases of dental caries (Laksmiastuti et al., 2019). Untreated decay may lead to a loss of primary teeth which is common, but seldom untreated leading to a widespread but under-recognized issue of early tooth loss and malocclusion in children (Ali et al., 2022).

Urban regions like Jakarta and Surabaya have proactive dental care due to both the availability of paediatric dental specialists and better parental literacy (Dewanto, Koontongkaew & Widyanti, 2020). However, more rural and remote provinces, such as Papua, East Nusa Tenggara, and Kalimantan, have the opposite issue: a large majority of parents do not even know or understand what space maintainers are, not to mention acknowledging the importance (Parker & Sudibyo, 2024). Moreover, many parents impose traditional beliefs and taboos, while others simply believe in “waiting for the permanent teeth to come” as if that waits away any neglect (Maida et al., 2015).

It is important to acknowledge the significant lack of research regarding knowledge of space maintainers in South East Asia (AlMotawah et al., 2022). While clinical-based studies have been conducted in India (Balakrishnan & Vadakkepurayil, 2023), Saudi Arabia (Redwan et al., 2024), and Turkey (Tuğutlu & Yılmaz, 2024), these studies have not offered solutions to current regional needs and contexts in Southeast Asia.

Parents’ perceptions play a powerful role in determining whether their children receive timely dental treatment. When early tooth loss is ignored or thought of as simply being replaced as it should, many restoration options are overlooked, including space maintainers. Addressing these misconceptions is key to ensuring preventive care is pursued when necessary (Steinvik, Svartdal & Johnsen, 2023). Furthermore, if parents are unaware of the need for functional or orthodontic services regarding premature loss of a tooth, they may opt out of, or deliberate delaying needed intervention based on unqualified perception that “a new tooth will come anyway” (Alfuriji et al., 2025). Parents must be aware and responsible for their children’s health care-seeking behaviour to lay the groundwork for oral health for their lifetime (Kaushik & Sood, 2023).

This complexity is made more difficult given the range of media available in Indonesia. Since smartphones and the internet are more accessible, many parents utilize social media, online forums, or unregulated health websites for information about dental health, which all have their risk of misinformation (Sasikala et al., 2025). They are also more likely seeking professional advice from dentists only in emergencies rather than preventive counselling (Shahin et al., 2025).

In the contextual examination of parental health behaviors in Indonesia, it is essential to consider the extensive geographical and cultural diversity across its 17,000 islands and over 300 ethnic groups (Widayanti et al., 2020). Factors such as varying educational levels, socioeconomic status, urban and rural distinctions, and access to dental care must all be taken into account when developing oral health programs and policies (Luo et al., 2021). Assuming there is uniform awareness among parents or applying a model based on behaviour from another country in this situation may lead to poor or unanticipated outcomes. Additionally, national dental health programs in Indonesia such as the Ujian Kesehatan Gigi Sekolah (UKGS) (School Dental Health Programme) are focused on education on brushing habits and caries prevention and not space maintainers and the education around interceptive orthodontics (Chairunisa et al., 2024). Nonetheless, this experience has created a gap both educationally for parents and clinically for clinicians providing these services.

On the above background, this national cross-sectional study aimed to determine the knowledge, attitudes, and sources of information of Indonesian parents regarding pediatric space maintainers (PSM) and to explore socio-demographic predictors of their knowledge and attitudes. The findings will be useful for developing targeted approaches to education, helping to inform the development of preventive orthodontic policy, and a means to build upon dental education curricular.

## Materials & Methods

### Ethics approval

This study was conducted following ethical guidelines and was approved by the Universitas Airlangga Faculty of Dental Medicine Health Research Ethical Clearance Commission. The approval reference number is 723/HRECC.FODM/VII/2023, dated July 10, 2023. Participants’ participation was entirely voluntary, and they were free to leave the research at any moment. The respondents gave their consent before the commencement of their responses to the survey to use them in the current study without any personal identification.

### Study design

This research employed a descriptive cross-sectional study to evaluate the awareness, attitudes, and information sources among Indonesian parents on the use of space maintainers in paediatric dentistry. The cross-sectional design allowed a snapshot evaluation of parental views, facilitating an assessment of current levels of parental awareness and perception at a point in time.

### Study population and sampling

The study population was made up of Indonesian parents and caregivers of children ages 3–12 years, living in urban, semi-urban, and rural areas in Indonesia. Participants were recruited from public and private dental clinics, schools, and social media accounts so that they would be representative of various geographic and socio-economic contexts while also accounting for differences in case management due to factors including culture and accessibility to healthcare services. A stratified positional multi-stage cluster sampling approach was followed so that participants were representative of these contexts. The minimum required sample size was calculated using Cochran’s formula for proportions under the conservative assumption of a population of 1 million, a predicted proportion of 74% from previous studies, an estimated margin of error of 5% and a 95% confidence level. Based on a stratified group, this led to a sample size of 296 that was adjusted to take into consideration the design impact of 1.

### Inclusion and exclusion criteria

#### Inclusion criteria

• Parents or caregivers of children aged between 3 to 12 years old.
• Residents of Indonesia who provided informed consent for participation in the current study.
• Parents who had visited with their children for a dental consultation within the last year.

#### Exclusion criteria

• Dental professionals or individuals with formal dental training (to avoid bias due to prior knowledge).
• Non-residents of Indonesia.
• Parents of children undergoing active orthodontic treatment, to avoid skewed responses due to ongoing intervention experiences.

#### Questionnaire development

The questionnaire consisted of 16 questions and consisted of four sections. The questionnaire contained both multiple-choice and open-ended questions. The sections of the questionnaire are as follows:

• Demographic information of parents.
• Parental knowledge about space maintainers.
• Parental attitudes toward space maintainers.
• Parents’ sources for information on space maintainers.

### Section 1: demographic details of parents

In this section, we collected demographic data, such as the participant’s age and gender, as well as information on the parents’ educational backgrounds. Data on family size, including the number of children and their ages, was also acquired. One question revealed information about how frequently parents arranged dental checkups for their children.

### Section 2: parental knowledge about space maintainers

This section constituted a pivotal aspect of our questionnaire, focusing on gathering data regarding the number of parents who were aware of paediatric space maintainers. Parents who indicated awareness were subsequently directed to proceed with the survey, allowing us to gather insights into their knowledge of space maintainers. Questions about utilization and various types pf space maintainers were included in the survey. Additionally, parents were enquired whether they thought space maintainers were a common treatment.

### Section 3: parental attitudes toward space maintainers

This particular section focused on gathering information about parental attitude towards space maintainers. Participants were asked to rate their comfort level, which ranged from ‘very comfortable’ to ‘very uncomfortable’. They also cited reasons for their comfort and discomfort levels, such as cost, discomfort to children, belief in the dentist, and fear of complications. Also, parents were asked whether they considered alternatives like braces or other treatments instead of space maintainers.

### Section 4: sources of information for parents on space maintainers

This section provided input on the sources parents utilize to acquire knowledge about their child’s dental health, such as whether they discussed space maintainers with their dentists or actively sought information from online parenting forums or platforms. One question explored the impact of advice and experiences shared by other parents on their decision-making process.

This cross-sectional study targeted the population of either current parents or prospective parents residing in who were recruited from social media platforms. The questionnaire was designed using Google Forms and widely distributed among parents through social networking sites, primarily WhatsApp, Telegram, and Instagram. Although this method enabled broad and rapid dissemination, there also existed the possibility of selection bias since some parents who are not digitally literate or do not have reliable internet access may have not be adequately represented. This is a particular limitation for lower income and rural populations as they tend to use community channels to gain health knowledge compared to digital channels.

### Validation of the questionnaire

A thorough examination of the literature and professional advice from two researchers with experience in survey methodology and two paediatric dentists served as the foundation for the questionnaire’s creation. The knowledge items were adapted from Visanth et al. (2024) and Linjawi et al. (2016) while the attitudinal constructs were adapted from frameworks by Alduraihim et al. (2020) and Ali et al. (2022). The sources of information were guided by Doğan, Polat & Özüdoğru (2025). Furthermore, the demographic items and questions related to culturally specific matters were newly developed to reference the Indonesian context.

Each item was examined by a panel of experts to ensure that it was relevant and in line with the desired constructions in order to establish content validity. In a pilot study, 30 parents rated the questions’ comprehensibility and clarity in order to determine face validity. The appropriate changes were made to improve clarity and reduce ambiguity in response to their input. Factor analysis was used to confirm that the items were logically grouped within the predetermined categories of knowledge, attitudes, and information sources in order to assure construct validity. Questions with factor loadings below 0.4 were either revised or excluded to strengthen the overall validity of the instrument.

### Reliability of the questionnaire

Cronbach’s alpha was used to assess the questionnaire’s internal consistency; the result was 0.82, suggesting a high degree of reliability. By giving the questionnaire to 30 participants twice over a two-week period, test-retest reliability was evaluated. The results showed strong stability over time, with an intraclass correlation coefficient (ICC) of 0.89. With a 92% agreement rate between two independent reviewers, inter-rater reliability was also assessed. These metrics attest to the questionnaire’s ability to deliver consistent and trustworthy data for evaluating parents’ attitudes, knowledge, and information sources on space maintainers.

### Statistical analysis

The authors entered data into Microsoft Excel and examined it using IBM SPSS Statistics version 26 (IBM Corp., Armonk, NY, USA). The study relied on descriptive statistics (frequency, percentages, mean, and standard deviation) to summarize participants’ demographic characteristics and responses regarding knowledge, attitudes, and sources of information about space maintainers and used inferential statistics to test for associations and predictors. For categorical variables (*e.g.*, place of residence, education level, prior dental visits), Chi-square (*χ*^2^) tests were used to check for relationships with key outcomes (knowledge and attitude levels). For comparing means across continuous or ordinal variables, independent-sample t-tests and one-way analysis of variance (ANOVA) were used when appropriate. Binary logistic regression analyses were performed to test possible predictors of significant parental awareness and attitude towards space maintainers. The authors conducted *post hoc* interaction analysis (Knowledge × Residence) to test for moderation effects. All models were controlled for relevant covariates (including education, income, and frequency of dental visits). Prior to regression analysis, the authors ran multicollinearity diagnostics. Variance Inflation Factor (VIF) scores for all predictors were under 2.0, and tolerance levels were higher than 0.1, indicating acceptable levels of multicollinearity. To assess the model fit, the authors looked at the Hosmer–Lemeshow goodness-of-fit test. All models had non-significant results indicating acceptable fit to the observed data. A *p*-value of less than or equal to 0.05 was considered statistically significant.

## Results

### Demographic profile of respondents

**Figure 1:**
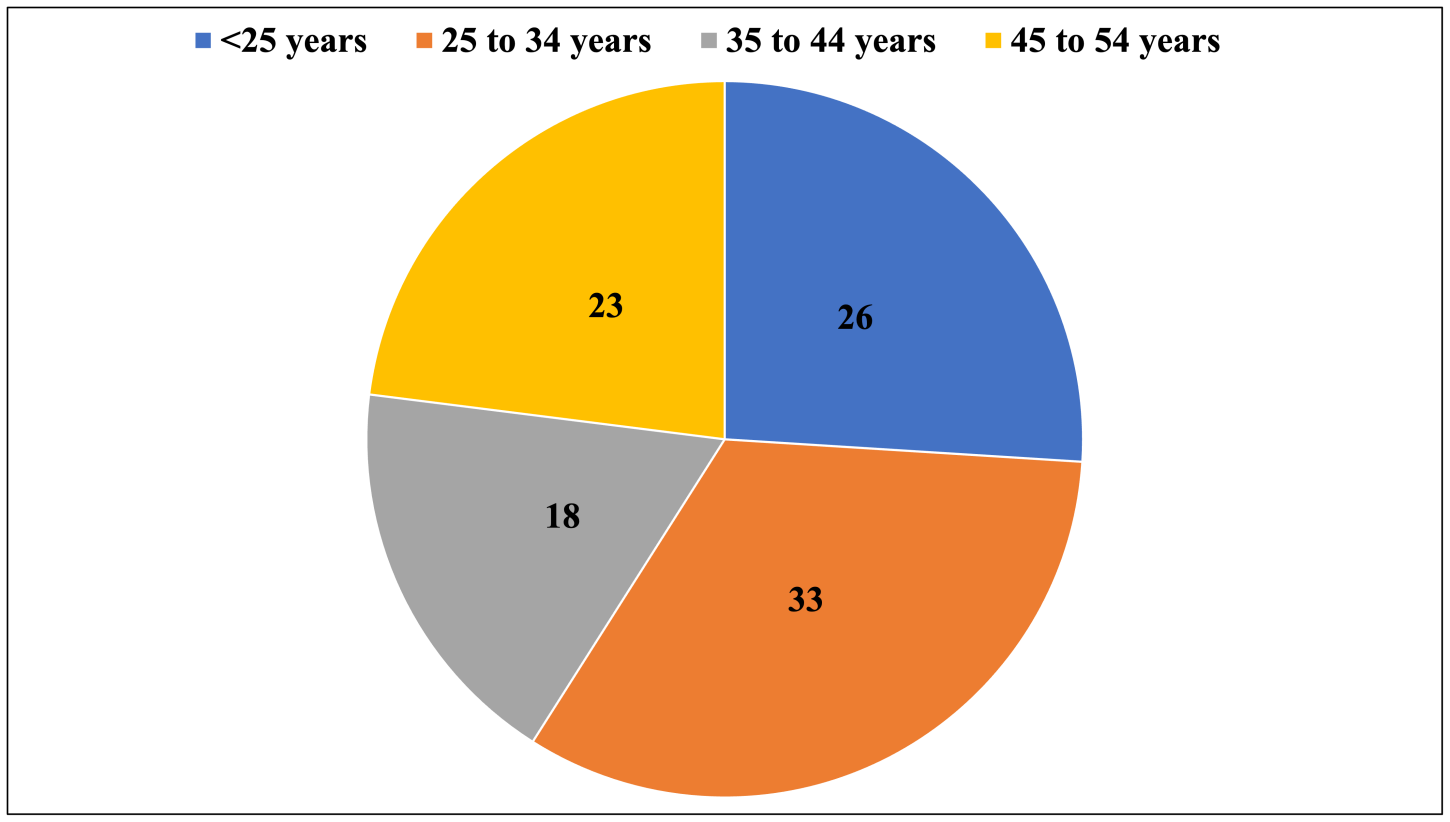
Distribution of the respondents according to the parents age.

**Figure 2:**
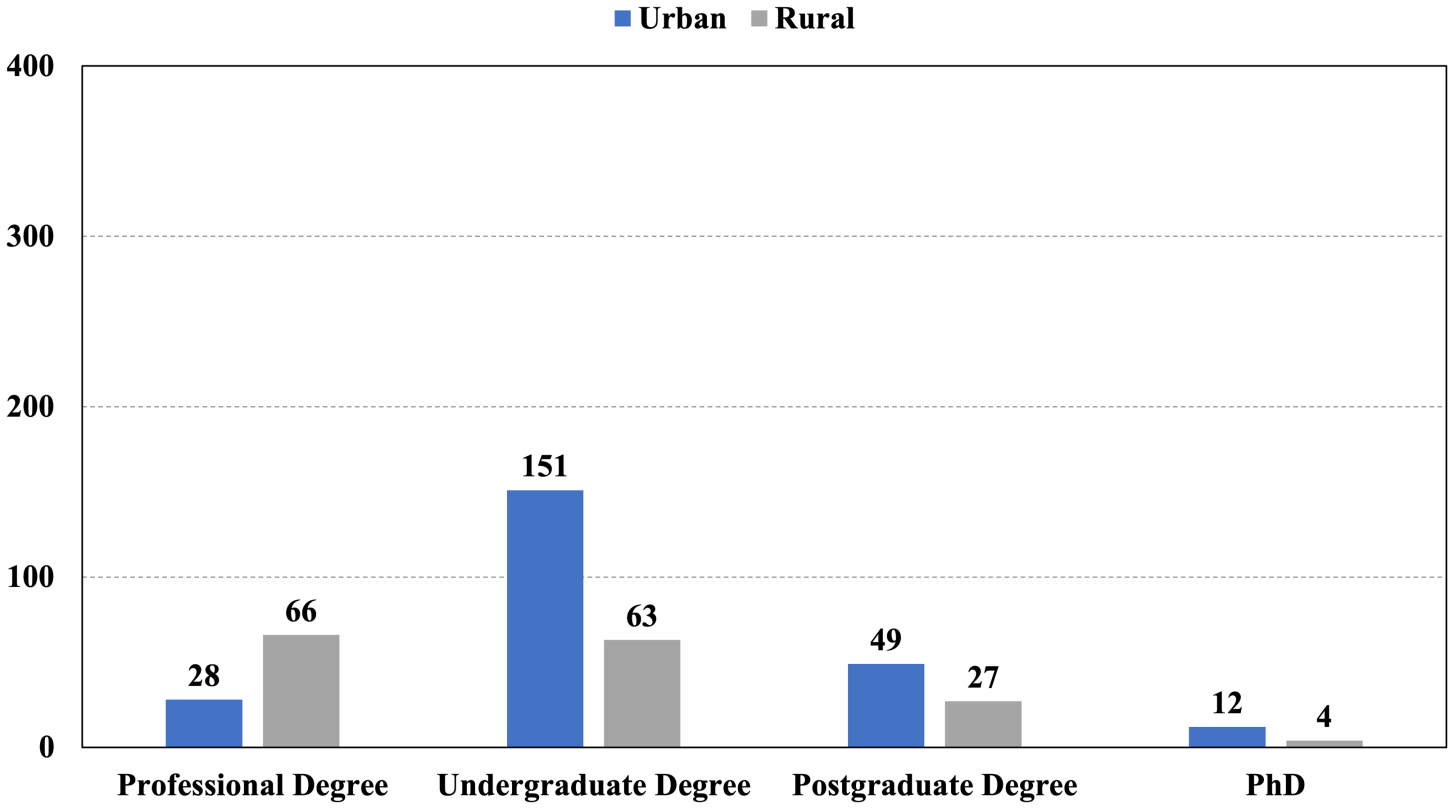
Distribution of the respondents according to the parents age and their educational degree.

The final sample consisted of 400 Indonesian parents, overwhelmingly distributed in urban locations (*n* = 240; 60%) over rural ones (*n* = 160; 40%). The majority of respondents identified as female (73%), in line with current demographic patterns of child health research, which indicates that mothers are still more commonly the primary caregivers and decision-makers, specifically in paediatric health. The age distribution was as follows: <25 years (26.0%), 25 to 34 years (33.0%), 35 to 44 years (18.0%), and 45 to 54 years (23.0%) (Fig. 1). The composition of participants’ families varied, with 33.75% reporting no children, 31.75% having one child, 30.75% having two children, and only 3.75% reporting three or more children. Education had a marked cross-tabular difference by location among respondents (*χ*^2^ = 35.2, *p* < 0.001) (Fig. 2). Metropolitan parents tended to have undergraduate degrees or higher, suggesting differing access to education which may have impacted access to health literacy.

### Dental visit patterns by residential location

**Figure 3:**
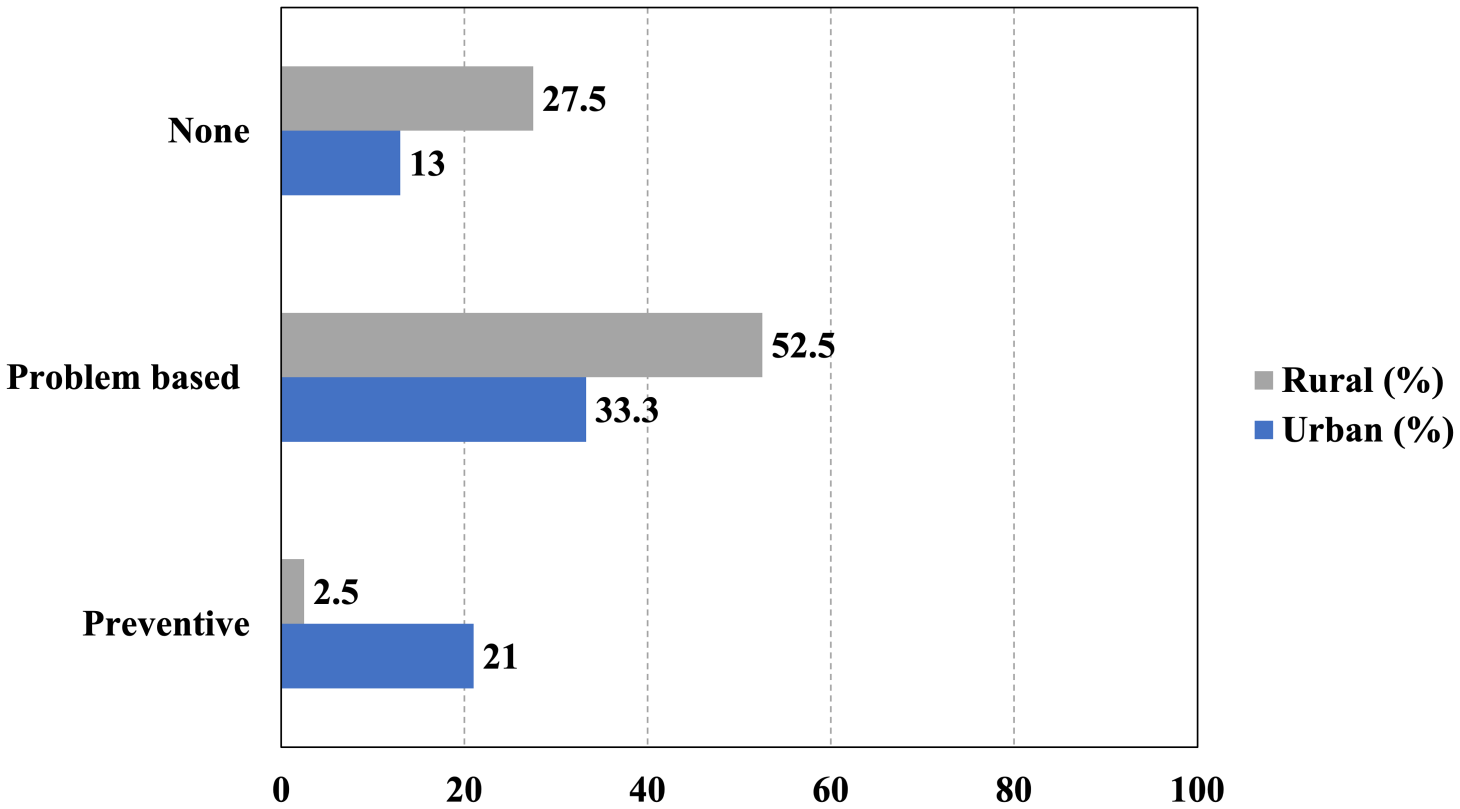
Distribution of the respondents according to the dental visit patterns.

A significant disparity in access to paediatric dental care is evident when comparing urban and rural populations, highlighting a critical public health issue. Specifically, only 21% of urban children have ever received preventative dental care, whereas a mere 2.5% of rural children have had the same experience. This difference is statistically significant (*χ*^2^ = 38.9, *p* < 0.001) and is nearly ten times different and indicative of structural inequities in the delivery of oral health services. A further look at the parental reports of the types of visits indicate more blatant differences; medically-motivated visits (*i.e.,* visits related to pain, infection, or visual decay) occurred at a higher rate for rural children (52.5%) than urban children (33.3%) in part because of a primarily reactive and not preventative strategy to oral health for rural visits in conjunction with the sad fact that 27.5% of rural children have never been to the dentist (that’s more than twice as many urban children (13%) (Fig. 3). The result points to a disparity between rural-urban access of preventative dental care for Indonesian children as being a structural inequity in the health care system in Indonesia.

### Preventative dental visit disparity

**Figure 4:**
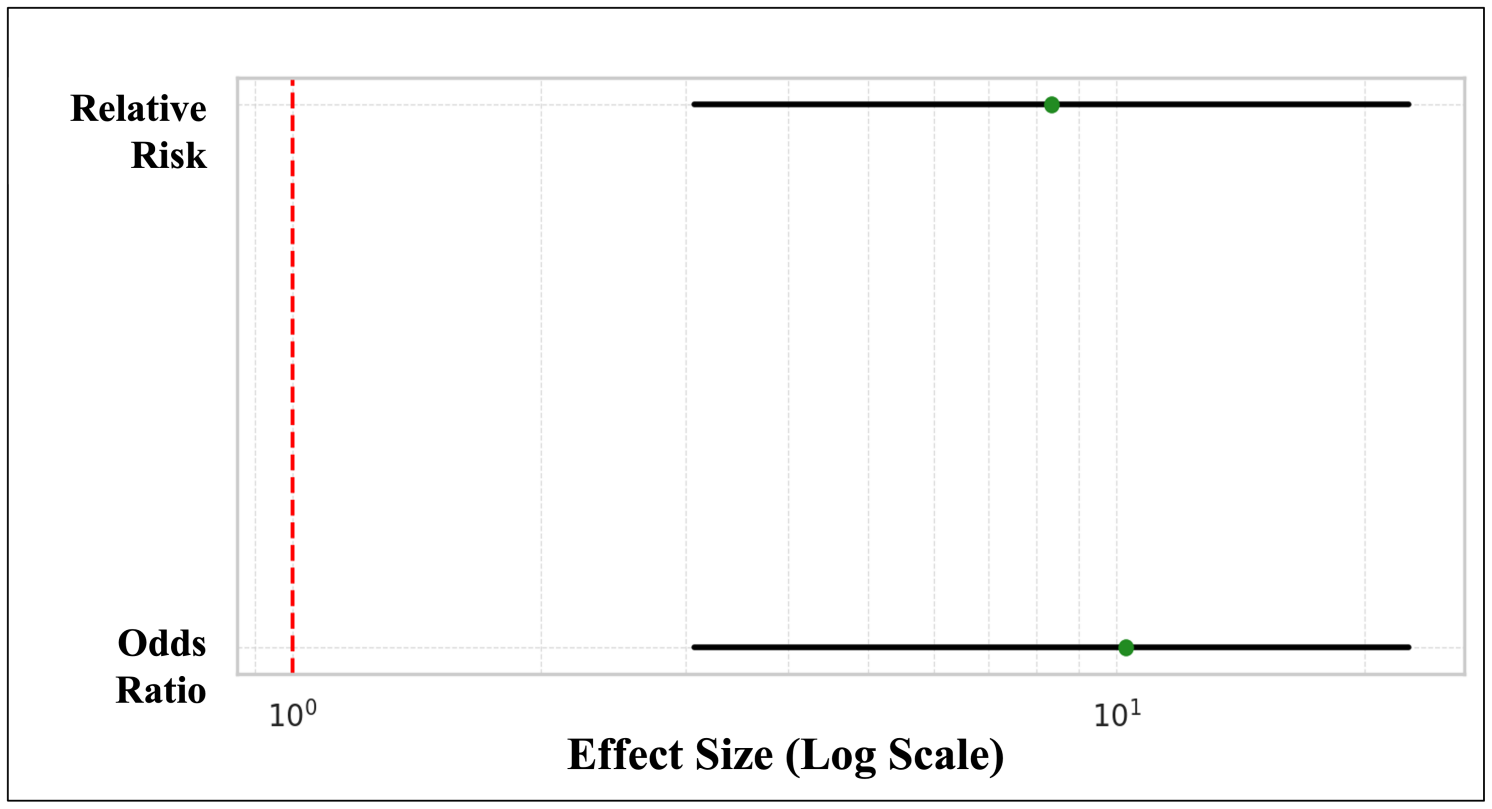
Disparity in preventative dental visits between urban and rural children. The red dashed vertical line at 1.0 represents the null effect. Since both confidence intervals lie well to the right of this line, it confirms a statistically significant and large effect—urban children are substantially more likely to receive preventative dental care.

A notable disparity in preventative dental visits was identified between urban and rural children. Specifically, 21% (50 out of 240) of urban children had received a preventative dental visit, in contrast to a mere 2.5% (four out of 160) of rural children. This difference was statistically significant, as demonstrated by a chi-square test (*χ*^2^ = 26.08, *p* < 0.001) and Fisher’s exact test (*p* < 0.001). The association between residential location and the receipt of preventative care was moderate, as indicated by Cramér’s V of 0.255. Urban children were 8.33 times more likely to have received preventative care than their rural counterparts (Relative Risk = 8.33, 95% CI [3.07–22.62]). The odds of receiving preventative care were over tenfold higher in urban settings (Odds Ratio = 10.26). Furthermore, the prevalence confidence intervals—21.0% (95% CI [16.2%–26.4%]) for urban children and 2.5% (95% CI [1.0%–6.3%]) (Fig. 4) for rural children—do not overlap, underscoring the strength and robustness of the observed disparity.

### Knowledge assessment

**Figure 5:**
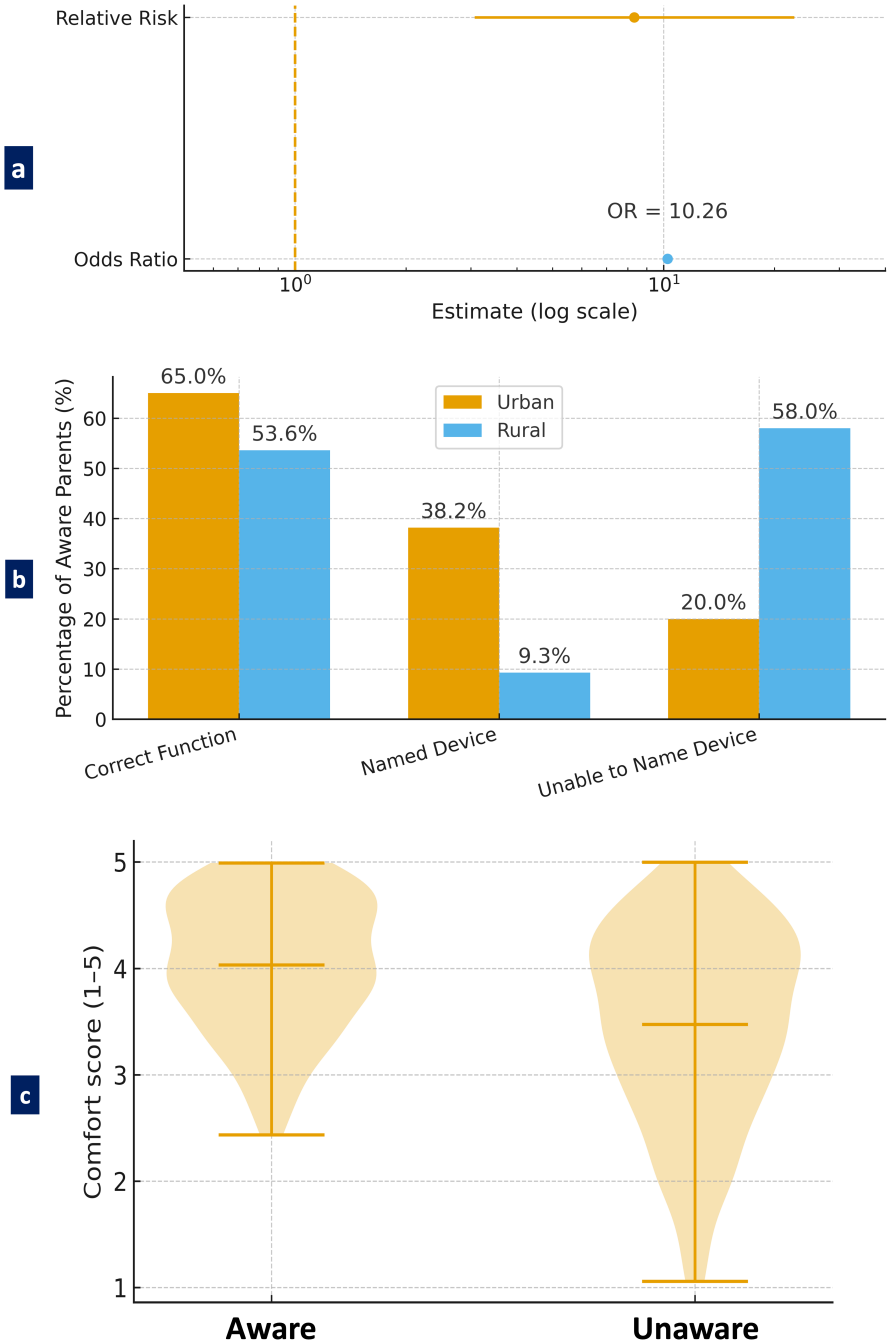
Parental knowledge and comfort regarding space maintainers. (A) A forest plot showing odds ratio and relative risk of space maintainer awareness between urban and rural parents, (B) A bar graph comparing depth of knowledge about space maintainers between urban and rural parents who reported awareness, and (C) A violin plot showing the distribution of parental comfort level with space maintainers based on awareness.

Of the participants, 25% (*n* = 100/400) indicated that they were aware of space maintainers. There was a statistically significant difference in awareness by location, as urban parents showed a much higher awareness (37.5%; 90/240) compared to rural parents (6.3%; 10/160). *χ*^2^ = 48.35, *p* < 0.001. The odds ratio was calculated at 10.26, and the relative risk was 8.33 (95% CI [3.07–22.62]), indicating that urban parents were over eight times more likely to be aware than their rural counterparts (Fig. 5A).

Of those who were aware, 65% correctly identified the functional aspect of space maintainers; that is, it mainly preserves space in regards to teeth that erupt permanently and that it does not promote malocclusion. However, it was clear that the quality of knowledge was inconsistent, as 46.4% of the rural parents who were aware offered vague or incorrect descriptions, suggesting a gap in functional understanding. Furthermore, there were notable deficits in device-specific knowledge: 38.2% of aware urban parents could name appliances such as the band and loop, compared to only 9.3% of rural aware parents; 58% of the latter could not name any device (Fig. 5B). Parents’ level of comfort with space maintainers was significantly impacted by their level of awareness. Parents who were aware of space maintainers reported a higher level of comfort (*M* = 4.21, SD = 0.91) than parents who were unaware of space maintainers (*M* = 3.76, SD = 1.12) and this difference in comfort level was statistically significant (t(283) = 3.26, *p* = 0.0013). The distribution of comfort scores based on awareness was shown in Fig. 5C, which consistently demonstrated a positive relation between knowledge and comfort.

### Attitude assessment

**Figure 6:**
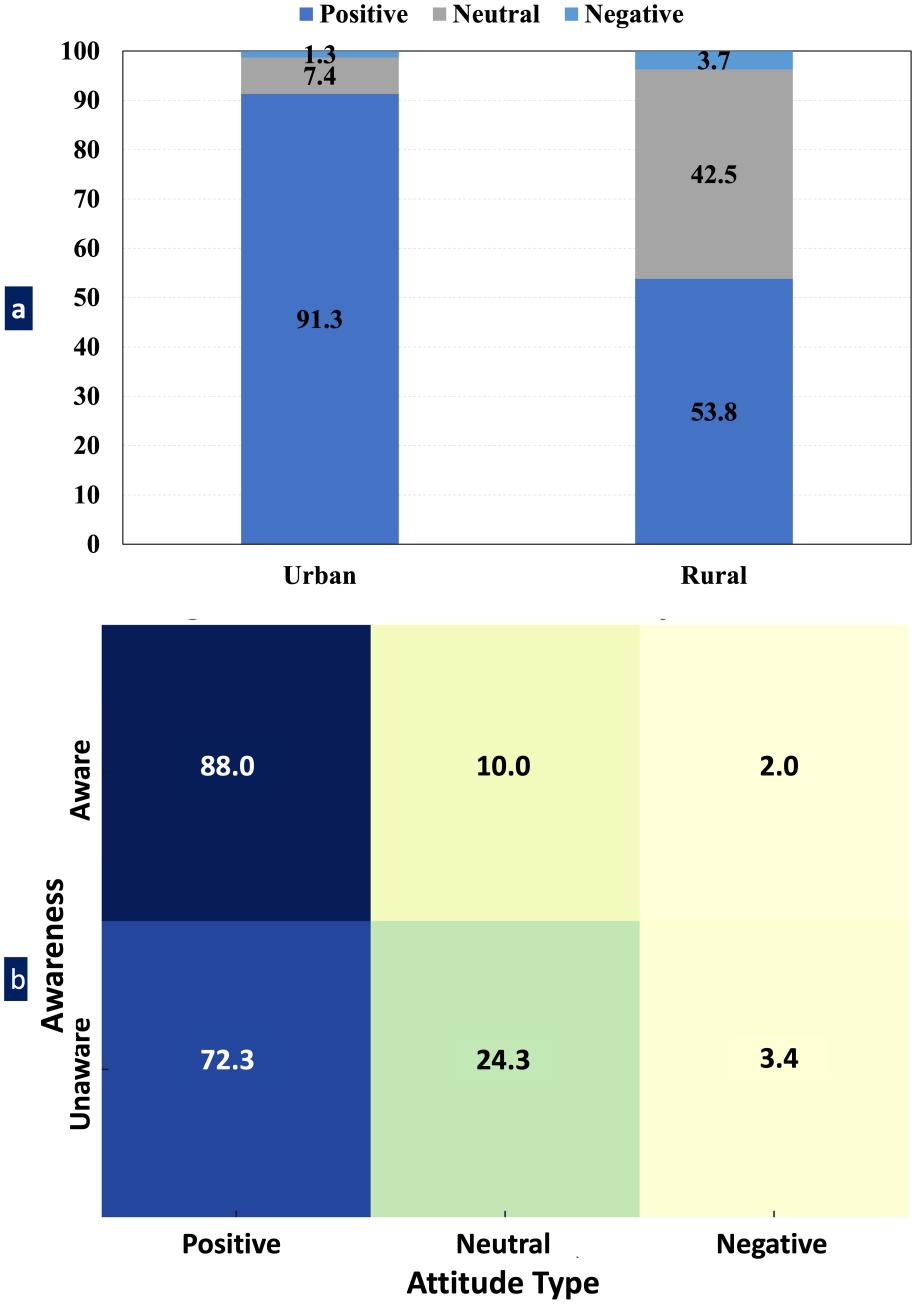
Attitude distribution toward space maintainers by awareness and residence. (A) Stacked bar chart illustrating the distribution of parental attitudes (positive, neutral, and negative) toward space maintainers based on geographic location (urban *vs.* rural) and (B) Heatmap showing the relationship between parental awareness of space maintainers and their attitude.

Parental attitudes toward space maintainers exhibited significant variation based on geographic location. Among urban parents, 91.3% demonstrated positive attitudes, in contrast to 53.8% of rural parents. Rural respondents were more frequently neutral (42.5%), with only a small minority (3.7%) expressing negative attitudes. In contrast, only 7.9% of urban parents expressed neutrality, and less than 1% expressed a negative attitude (Fig. 6A). This difference was statistically significant (*χ*^2^ = 74.58, *p* < 0.001). Being aware of space maintainers also showed to be an important predictor of positive attitude: while 88% of parents who were aware expressed a positive attitude toward space maintainers, only 72.3% parents who were not aware expressed a positive attitude. Urban parents among the aware parents were very favourable (92.2%), while aware parents residing in rural regions were less favourable (50%). Likewise, the association between awareness and attitude was statistically significant (*χ*^2^ = 10.88, *p* = 0.0043). Figure 6B presents a detailed cross-tabulation of awareness and attitude responses showing that the unaware group had a higher rate of neutral and negative responses. These findings provide insight into the importance of front-end education and awareness of preventive dental treatment to develop positively enhanced attitudes.

### Interaction between knowledge and residence

A logistic regression analysis was performed to examine the interaction effect between knowledge (awareness) and residential area on parents’ positive attitudes. The results indicated that urban residence significantly predicted a higher likelihood of a positive attitude (adjusted odds ratio (AOR) ≈ 8.27, *p* < 0.001). However, neither knowledge alone nor its interaction with residence was statistically significant (*p* = 0.806 and *p* = 0.658, respectively) (Table 1). These findings suggest that geographic location exerted a stronger influence than awareness alone, and that awareness did not significantly moderate the effect of residence on attitude within this model.

**Table 1:** Logistic regression with knowledge × residence interaction term. Multivariate logistic regression analysis was performed to assess associations between parental awareness and predictors. Interaction terms were included to evaluate combined effects.

Predictor	AOR	95% CI	*p*-value
Knowledge (Aware)	0.85	0.24–3.07	0.806
Residence (Urban)	8.27	4.38–15.65	<0.001
Knowledge × Residence	1.43	0.29–7.04	0.658

**Notes.**

AOR, Adjusted Odds Ratio; CI, Confidence Interval.

### Information sources

The primary source of information for urban parents was the dentist, as reported by 68.75% of respondents. In contrast, only 41.88% of rural parents identified dentists as their source of information, a difference that is statistically significant (*χ*^2^ = 27.37, *p* < 0.001). Importantly, 25% of rural parents identified community sources as the source in which they heard about space maintainers, whereas no urban parents did (*χ*^2^ = 63.92, *p* < 0.001). Plus, 26.25% of rural respondents and 29.17% of urban respondents identified the internet or social media as potential sources (Table 2). These findings highlighted differences in sources of information, such that rural parents tended to rely on community-based sources, whereas urban parents primarily relied on the professional healthcare system (dentists).

### Associations and predictive analysis

**Figure 7:**
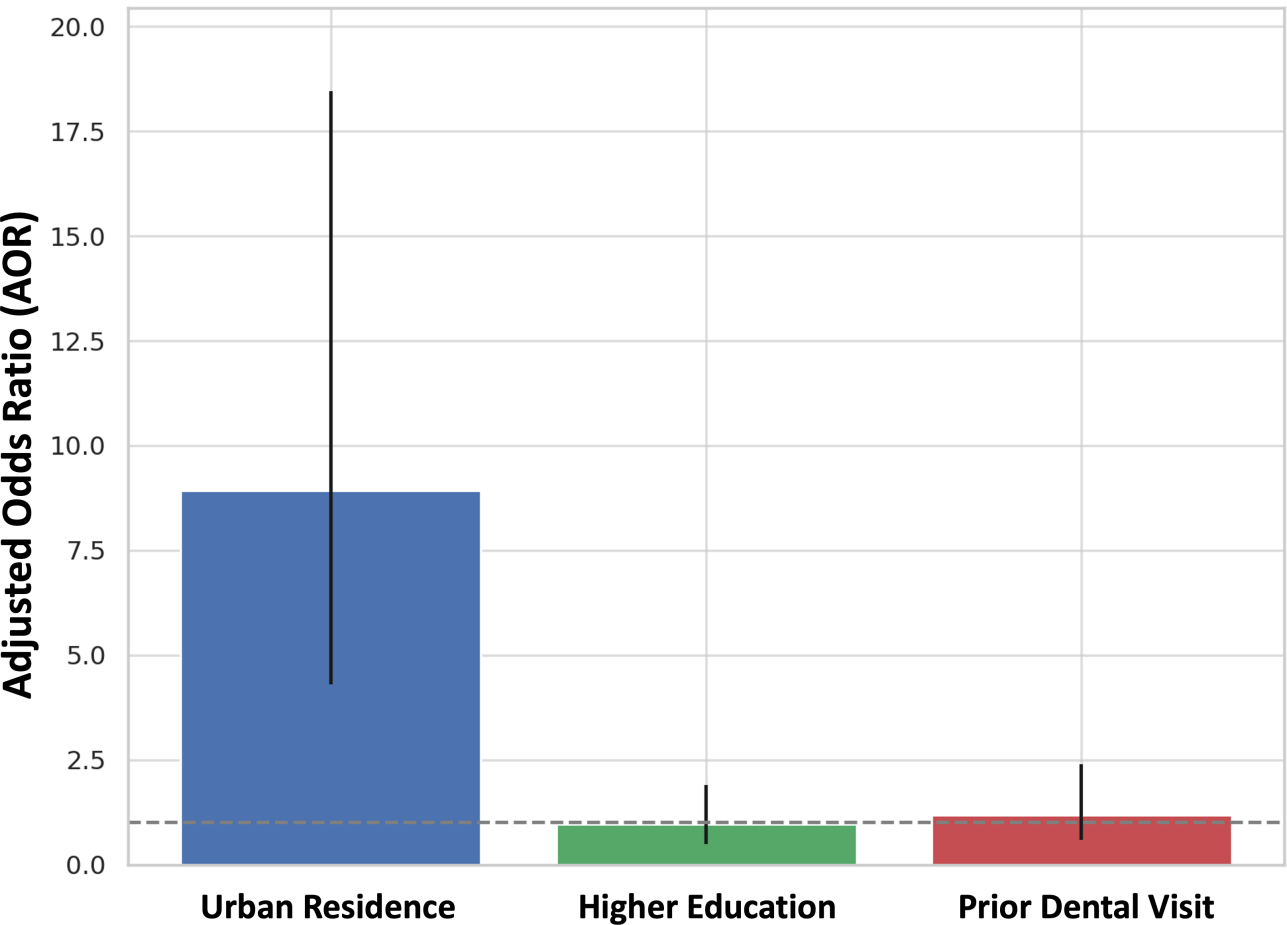
Bar chart displaying the adjusted odds ratios (AOR) from a multivariate logistic regression analysis assessing predictors of parental awareness of space maintainers.

Multivariate logistic regression analysis yielded urban residence as the sole significant predictor of awareness of space maintainers (AOR = 8.92; 95% CI [4.31–18.45]; *p* < 0.001). Higher education (AOR = 0.96; *p* = 0.897) and prior dental visits (AOR = 1.19; *p* = 0.632) did not reach statistical significance (Fig. 7). As a result, residential location is the best predictor for parental awareness, and urban parents are approximately nine times more likely to be aware than rural parents.

### Model diagnostics and assumptions

Prior to the multivariate logistic regression, we assessed for multicollinearity among the predictors by using Variance Inflation Factor (VIF). All the VIFs were less than 2.0 indicating that multicollinearity is not a problem, and all tolerance statistics were above the suggested cutoff value of 0.1 indicating acceptable levels of independence between all predictors.

The fit of each model was assessed using the Hosmer–Lemeshow goodness-of-fit test. The model quantifying predictors of awareness of space maintainers was not significant (*χ*^2^ = 5.21, *df* = 8, *p* = 0.736) and indicated a good fit to the observed data. The attitude model produced similar findings (*χ*^2^ = 6.84, *df* = 8, *p* = 0.552) which suggest that both logistic models successfully captured the observed or latent patterns in these underlying data.

**Table 2:** Source of information about space maintainers among urban and rural parents.

Source of information	Urban (%)	Rural (%)	*χ* ^2^	*p*-value
Dentist	68.75	41.88	27.37	<0.001
Community members	0.00	25.00	63.92	<0.001
Internet/Social-Media	29.17	26.25	0.27	0.6011

**Notes.**

Percentages represent the proportion of parents citing each source. *χ*^2^ = Chi-square test was applied to assess differences between urban and rural groups.

## Discussion

The national survey found a significant knowledge gap on space maintainers among Indonesian parents, especially among those from rural areas. Parents were aware of space maintainers, although the awareness was variable in quality, and there were many misconceptions regarding the purpose or need for a space maintainer.

Our findings match and complement the evolving literature from both developed and developing nations. Research has consistently shown that parental knowledge of space maintainers is considerably low, despite the increasing options for paediatric services (Alzubaidi, 2024; Visanth et al., 2024; Sajadi et al., 2024; Doğan, Polat & Özüdoğru, 2025). Generally, less than 30% of parents, attending paediatric dental outpatient departments, are aware of the existence of space maintainers and their potential preventative aspects (AlMeedani et al., 2020). Also, well-informed parents adhered to misconceptions regarding the need to replace prematurely lost primary teeth (Gomes et al., 2022). Comparable patterns have been observed in neighbouring Southeast Asian countries, that identified a lack of parental understanding regarding interceptive orthodontics, which was attributed to insufficient public health promotion in paediatric dental care (Alfuriji et al., 2025). In the Middle East, research has highlighted the significant influence of cultural perceptions, with many parents considering primary teeth as temporary and not warranting restoration or maintenance (Alharbi, Alharbi & Kolarkodi, 2022; Mohammed Alwusaybie et al., 2024).

Cultural beliefs about primary teeth being “disposable” or that extraction is enough following decay have been identified in both rural and urban communities. Families often just extract a decayed or mobile tooth without realizing the orthodontic and developmental effects of losing space (Masri et al., 2025). Financial reluctance to invest in an intervention (especially among lower socio-economic groups) compounds many of these issues (Raison, 2019). Our results corroborate international research that found a significant lack of structured parental counselling at dental visits. It is well-established that in many situations general dental practitioners ignore or poorly explain the need for space maintainers, particularly in busy clinical contexts (Albati et al., 2018). This situation again reinforces the need to develop evidence-based chairside communication protocols that are structured to support the anticipatory guidance and communication of preventive care messages (Featherstone et al., 2021).

Importantly, knowledge is not sufficient for preventive action. In our study, even parents that regularly used space maintainers were taken to the dental office rarely for preventive visits, demonstrating a divergence between knowledge and practice. Societal and economic barriers, knowledge of treatment discomfort and preference for acute problems over preventive issues, most likely affect parents’ decisions related to their children’s oral health. Similar observations have been reported in oral health contexts, where knowledge is not associated with behaviour. Addressing the “knowledge–behaviour” gap requires interventions that lead to not only knowledge but also understanding of parental motivation, financial circumstances, and cultural health-seeking behaviours.

The findings highlight urgent public health priorities. The widespread dependence on informal or non-professional sources highlights systemic deficiencies in preventive dental education and trust in healthcare systems. We must first take an institutional approach to parental advice and education at the initial consultation, and this should happen during paediatric OPDs as well as general dental practice that service children. Training and motivation of the dental profession is also necessary to properly communicate and promote their utmost importance. A number of methods should be developed, including the use of visual aids, simplified terms, and culturally sensitive terms to communicate the importance of arch space maintenance (Sengupta, Chakrabarti & Mukhopadhyay, 2019; Ho et al., 2024). Importantly, this advice and discussion need to be formalised as examination and treatment plans as suggested standard operating procedures, away from a discussion model to be formalised in the medical/dental record as this demonstrates ‘this is something that you will be doing routinely’ *versus* a ‘nice to have’. Therefore, as dental health professionally we can act now to improve the dental health of generations to come.

Embedding space maintenance and other preventive measures in school-based oral health programs for children and parents is critical. When dental health checkups are offered at school, when there are workshops for parents or information sessions for parents’ role in ensuring a child’s best health or ensuring early detection, who does not want to talk to their local dentist about caries prevention, early orthodontic screening and oral hygiene, *etc*. Preventive dental care and its positive effects on a child’s overall craniofacial development are not only important to understand, but we need to use these opportunities to educate and discuss oral care from parents and children alike (Duijster et al., 2015). Public health campaigns to dispel misconceptions about primary teeth are also crucial, especially in rural and underserved areas. Public health campaigns to stress the importance of beginning to ensure early and preventive care in children’s lives from the very start is key in rural and underserved areas (Prowse et al., 2014). Perhaps using mobile clinics; community event outreach; community engagement about child oral health on the part of various levels of government; etc. It is also important to utilize tenants of misinformation—in other words—utilizing social media as it serves as a prominent source of misinformation, in thinking of strategies to commit to continuing develop verified, appealing, and multilingual content focused on parents.

The data discussed have significant policy implications. The UKGS is another national initiative focusing on oral hygiene and prevention of caries but not factors contributing to premature loss of tooth or interceptive orthodontics. Adding education on space maintainers (including interim prosthesis) to preventative strategies would increase the scope of prevention. A public health services requires upper level policy and services to promote the prevention of orthodontic problems not the orthodontic problem existene. Service-level policy also needs to revive dentist-parent communication guidance. Local goverements can even support their region based training modules to help their dentist and parent communication to improve prelim shared decision making in their community clinics. Public Health has potential to replace misinformation on social media with factual, multilingual, and culturally relevant information promoting preventative orthodontics and dentristy.

Closing the gap between knowledge and behavior involves interventions that incorporate strategies to promote both logistical and culturally based access. School collaboration workshops, for example, with outreach programs through mobile clinics, or community health centers, or parent-dentist sessions, can promote preventive education for these at-risk families. Programs to reduce access cost barriers using subsidized dental appliances, limit non-compliance access fears with demonstrations, and adapt’d messages to a culturally-acceptable context by acknowledging many common everyday practices and re-directing messages regarding the long-term risks related to early tooth loss. This kind of accessible and culturally relevant effort will more likely convert knowledge into preventive behavior.

This study has many unique strengths. It is one of the few national representative surveys that measure parental knowledge or understanding of space maintainers in Indonesia. The large sample size and geographic diversity also improve generalisability of our findings. The use of a psychometrically valid and piloted questionnaire with established internal consistency and valid findings, also added to our confidence in our results. We must acknowledge some limitations of this study. Firstly, cross-sectional designs limit our ability to assess causal relationships; the mentor-buddy relationships will need to be followed longitudinally to be able to assess real behavior changes following educational activities. Additionally, self-reported data could have recall bias and social desirability bias, particularly for those questions relating to attitudes or previous dental visits. Further, while efforts were made to include underrepresented populations, remote tribal and hilly areas may remain under-sampled and limit true national representation. In addition, the method of survey distribution itself may have led to bias. Recruiting through social media may have biased recruitment away from parents who tend to be younger, more educated, and more connected, while systematically omitting parents with fewer internet access opportunities, particularly parents living in rural, economically disadvantaged communities. This digital divide may be part of the reason for the difference in knowledge and attitudes of urban *vs.* rural parents. Future studies should, therefore, include a combination of online recruitment and community-based approaches, such as recruitment in schools, health centers, and community organizations, to better capture a more inclusive sample.

Future studies should focus on longitudinal studies to examine whether systematic parent counseling, and community education have the potential to increase acceptance and uptake of space maintainers. Qualitative designs, such as narrative interviews (in-depth interviews) or focus groups, would provide a better understanding of the cultural beliefs, socio-economic barriers and parental fears that influence a parent’s decision. Lastly, implementation research or using a quality improvement approach to study how established counseling protocols can transition during routine paediatric dental visits, including school-based models, or chair-side conversation, will be important for translating knowledge into lasting change.

## Conclusion

This study emphasizes the very common issue of under-developed parental awareness, and diverse attitudes towards the space maintainers in Indonesia. Even if awareness of oral health in children is improving, there are still misperceptions, especially with regard to the importance of space maintainers after the loss of an early tooth. A particularly critical finding was that parents residing in rural areas demonstrated markedly lower awareness than their urban counterparts, underscoring persistent inequities in access to preventive dental knowledge and services. The research highlights that dental professionals are seen as the most reliable and powerful sources of information; however, this potential is not being achieved as it is not being shared proactively and engaging with the public.

##  Supplemental Information

10.7717/peerj.20363/supp-1 Supplemental Information 1 Raw data

10.7717/peerj.20363/supp-2 Supplemental Information 2 Questionnaire

## Additional Information and Declarations

### Data Availability

The following information was supplied regarding data availability: The raw data is available in the Supplemental File.
