# Supplementary material for: Indonesian parental knowledge, attitudes, and sources of information regarding pediatric space maintainers: a cross-sectional questionnaire-based study
Source: PeerJ. 2026 Jan 5;14:e20363. doi: 10.7717/peerj.20363 (PMC12782031; doi:10.7717/peerj.20363)
Supplement: Supplemental Information 2 [file peerj-14-20363-s002.pdf]

Survey Questionnaire: Parental Knowledge and Attitudes About Pediatric Space Maintair

Section 1: Demographic Information

1. What is your age group?
   - Under 25
   - 25-34
   - 35-44
   - 45-54
2. What is your gender?
   - Male
   - Female
   - Non-binary
3. What is your highest educational qualification?
   - Undergraduate Degree
   - Postgraduate Degree
   - PhD
   - Professional Degree
4. How many children do you have?
   - 0
   - 1
   - 2
   - 3 or more

Section 2: Knowledge About Space Maintainers (Close-Ended)

5. Have you ever heard of a space maintainer?
   - Yes
   - No
6. What do you believe is the main purpose of a space maintainer? (Choose one)
   - To maintain space for permanent teeth to erupt
   - To correct crooked teeth
   - To reduce tooth decay
   - I don-t know
7. Which of the following are types of space maintainers you are aware of? (Select all that apply)

- Band and loop
- Crown and loop
- Lingual arch
- Distal shoe
- I have heard of space maintainers, but cannot name any
- I don-t know any types

8. In your opinion, how common is the use of space maintainers in children?
- Very common
- Somewhat common
- Rare
- I-m not sure

9. Do you think space maintainers are necessary if a child loses a tooth early?
- Yes, always
- Sometimes
- No, never
- I-m not sure

Section 3: Attitudes Toward Space Maintainers

9. How comfortable are you with using a space maintainer for your child?
- Very comfortable
- Somewhat comfortable
- Neutral
- Somewhat uncomfortable
- Very uncomfortable

10. If uncomfortable, what are your reasons? (Select all that apply)
- Cost
- Fear of complications
- Lack of knowledge
- Other (please specify)

11. Would you be willing to consider a second opinion if advised to use a space maintainer?
- Very willing
- Somewhat willing
- Not very willing
- Not willing at all

12. Would you consider any alternatives to space maintainers (e.g., braces)?
- Yes
- No
- Unsure

Section 4: Information Sources

13. Where did you first hear about space maintainers?
- Dentist
- Social Media
- Community
- Other

14. Have you discussed space maintainers with a dental professional?
- Yes
- No

15. Have you received information on this topic from other parents or forums?
- Yes
- No

16. Have you searched the internet to learn more about space maintainers?
- Yes
- No
